# Inclusion Complexes of Citronella Oil with *β*-Cyclodextrin for Controlled Release in Biofunctional Textiles

**DOI:** 10.3390/polym10121324

**Published:** 2018-11-29

**Authors:** Manuel J. Lis, Óscar García Carmona, Carlos García Carmona, Fabricio Maestá Bezerra

**Affiliations:** 1 Institute of Textile Research and Cooperation of Terrassa, Polytechnic University of Catalonia, C/Colom 15, Terrassa, 08222 Barcelona, Spain; oscargarciacarmona@gmail.com (Ó.G.C.); carlos.garcia.carmona@gmail.com (C.G.C.); 2 Textile Engineering, Federal University of Technology—Paraná, 635 Marcilio Dias St., Apucarana 86812-460, Parana, Brazil

**Keywords:** textile finishing, biopolymers, esterification, cotton, polyester, drug delivery

## Abstract

Biofunctional textiles with integrated drug-delivery systems can help in the fight against vector-borne diseases. The use of repellent agents derived from plants and oils is an alternative to DEET (*N*,*N*-diethyl-*m*-methylbenzamide), which has disadvantages that include toxic reactions and skin damage. However, some researchers report that oils can be ineffective due to reasons related to uncontrolled release. In this work, the mechanism of control of citronella oil (OC) complexed with *β*-cyclodextrin (*β*CD) on cotton (COT) and polyester (PES) textiles was investigated. The results obtained reveal that finishing cotton and polyester with *β*-cyclodextrin complexes allows for control of the release mechanism of the drug from the fabric. To assess the complexes formed, optical microscopy, SEM, and FTIR were carried out; the yield of complex formation was obtained by spectroscopy in the ultraviolet region; and controlled release was performed in vitro. Oil complexation with *β*CD had a yield of 63.79%, and it was observed that the release, which was in seconds, moved to hours when applied to fabrics. The results show that complexes seem to be a promising basis when it comes to immobilizing oils and controlling their release when modified with chemical crosslinking agents.

## 1. Introduction

Essential oils are chemicals made in nature that some plants use as a defense against certain species of insects. With the aim of replacing synthetic insect repellent agents, essential oils have been tested as protection against vector-borne diseases caused by insects [1,2].

An oil that has attracted attention because of its functionality and contribution is citronella essential oil, Cympobogon citratus [1,3,4,5]. Specos et al. [4] demonstrated that this oil shows repellency against mosquitoes, especially A. aegypty. On the other hand, some researchers have reported that this oil can be ineffective at controlling insects due to reasons related to uncontrolled release, i.e., it is desirable for the oil to be released in small amounts, prolonging the repellency for a long period of time. When released in large quantities, the duration of protection will be shorter. Therefore, how to control the release of these essential oils in textiles is a matter of great interest for researchers [6].

Many different essential oils have already been used in microencapsulation or complexation processes, among them lavender, rosemary, and jasmine, with medicinal and protective effects against vectors of disease [1,2,7] and protection against disease vectors [4,5,6]. These publications have demonstrated that the application of microencapsulated essential oils on textile substrates makes it possible to extend their lifespan, preventing fast evaporation. Biofunctional textiles are a new category of advanced materials combining conventional textiles with advanced systems of drug release based on biocompatible polymers [8,9]. The predominance of one of three mechanisms, Fickian, anomalous, or non-Fickian, invariably depends on the properties of the biofunctional textile used in the system, the geometry of the matrix, and the active principle [10].

Cyclodextrins (CDs) has been used for the functionalization of textiles [11,12]. The interaction between cyclodextrin and fibers has been reported in several works [13,14,15,16,17,18]. The use of poly(carboxylic) acids (PCAs), such as butane-1,2,3,4-tetracarboxylic acid (BTCA), as crosslinking agents allows a certain degree of fixation of the complexes formed.

**Figure 1:**
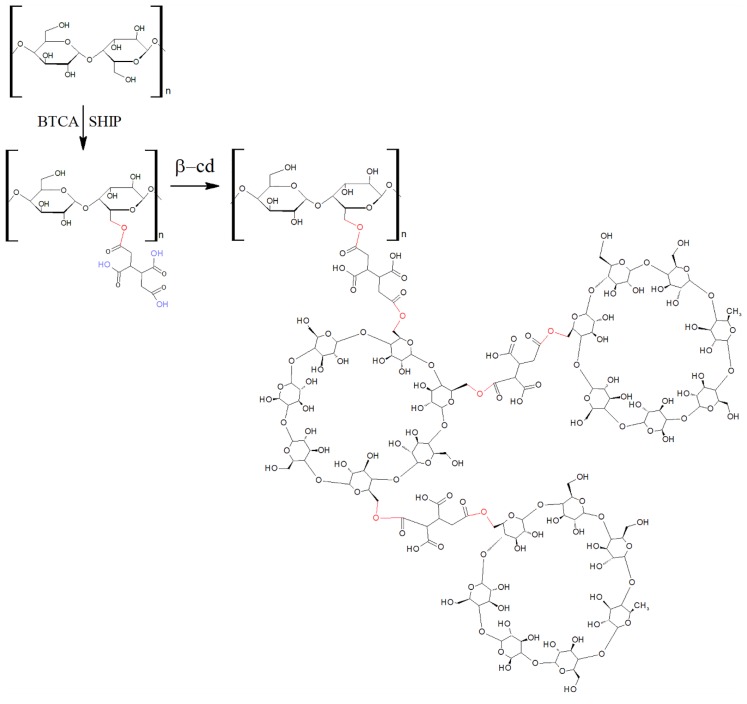
Grafting *β*-cyclodextrin (*β*CD) onto hydroxyl groups of cellulose via butane-1,2,3,4-tetracarboxylic acid (BTCA) as a crosslinking agent and the catalyst sodium hypophosphite.

As can be seen in Figure 1, BTCA has four carboxylic acid groups, which can react with the hydroxyl groups of cellulose by esterification [16,17,18], promoting *β*-cyclodextrin–cotton crosslinking. Heat or the salts of weak acids, such as sodium hypophosphite (SHPI), can act as catalysts of the esterification reaction [16].

**Figure 2:**
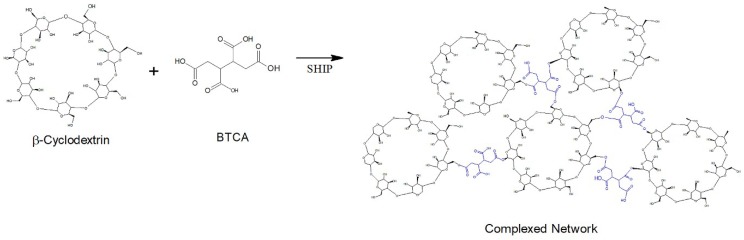
Complex network polymer formed between *β*CD and BTCA.

In the case of functionalization of polyester fibers, a complex network is covered by coating by the crosslinked polymer formed between the *β*-cyclodextrin (*β*CD) and the BTCA via a polyesterification reaction [19] (Figure 2).

Cyclodextrins are widely used in the complexation of pharmaceuticals [20,21], fragrances [22,23], flavorings [24,25,26], and oils [6,27,28,29] and as an absorbent of odors, oils, vitamins, caffeine, menthol, and biocides [23].

Bhaskara-Amrit et al. [30] emphasize that CDs play a key role in textile finishing and innovation. Their use provides immediate opportunities for the development of eco-friendly products with great potential for various applications. The increasing use of CDs in the textile area lies in processing [31,32] and finishing [2,25,27,30]. Textiles treated with CDs will be important for medical, hygienic, and home use [6].

The great interest in the application of CDs is that they can form inclusion complexes with volatile essential oils, protecting them against oxidation and improving their chemical stability [2,24]. Protection and controlled release are the biggest advantages, because they allow controlled release under the desired conditions, improving the effectiveness of the compound [33].

On the other hand, after the inclusion process, the active principle needs to be released. Costa and Lobo [34] report that the release of these encapsulated substances, in general, is accomplished by three mechanisms: diffusion, activation, and polymeric breakdown/erosion.

Quantification, as pointed out by Jain’s [35] study, can be obtained through in vitro assays using fixed parameters, which are correlated with the medium used. To this end, general equations are used, which present a dissolution ratio as a function of some parameters related to the polymeric form. 

## 2. Materials and Methods

Materials used to prepare the complexes were *β*CD, supplied by Wacker-Chemie GmbH, München, Germany, and citronella essential oil (WNFt, São Paulo-SP, Brazil). BTCA and SHPI, both provided by Sigma-Aldrich, São Paulo-SP, Brazil, were of analytical grade.

Standard woven fabrics, namely cotton fabric (bleached and desized cotton print cloth, style 400, 100 g m^−2^, ISO 105-F02) and spun polyester type 54 (style 777, 126 g m^−2^, ISO 105-F04) were used, both supplied by Thomas Scientific (Swedesboro, NJ, USA).

### 2.1. Preparation of Complexes in β-Cyclodextrin and Application

**Figure 3:**
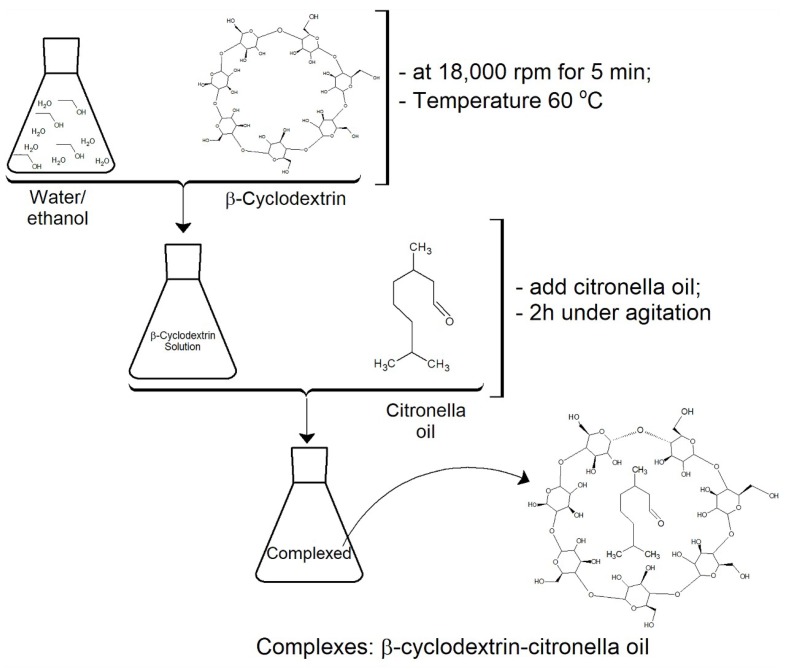
Experimental protocol followed for the formation of inclusion complexes CD–citronella oil.

A solution of 50 mL of ethanol and water (1:3) and 3 g of *β*CD was prepared and emulsified at 18,000 rpm for 5 min at 60 °C. High temperature and strong agitation allowed the cyclodextrin to become entirely soluble. Subsequently, 5 g of citronella oil was added. After 2 h of stirring, the complexes were formed. This is a standard process (see Figure 3) proposed by several authors, such as [2,26,36,37].

The application of the complexes onto the fabrics was done through a pad-dry process. This can be considered a green process, as it requires no organic solvent or toxic reactants [38]. Dehabadi et al. [39] proposed that the application should be followed by a curing step at 170 °C for 3 min to complete the process of fixation of complexes by crosslinking. Cotton and polyester textile articles were impregnated for 1 min in 6 g L^−1^ *β*CD solution, with BTCA 6 g L^−1^ used as the crosslinking agent and sodium hypophosphite 6 g L^−1^ as the catalyst, at room temperature for 3 min, as proposed by Vončina and Marechal [14]. Both substrates were submitted to the same conditions to see whether there was an influence of the chemical characteristics of the fibers, as stated in the hypotheses of the mathematical approach.

The crosslinking reaction proceeded via the esterification mechanism between the carboxylic groups of BTCA and the hydroxyl groups of cellulose and polyester, and a complex network was covered by coating the crosslinked polymer formed between the *β*CD and the BTCA (Figure 1 and Figure 2).

### 2.2. Characterization of βCD–Oil Complexes

A spectra of samples were examined in an FTIR instrument (Nicolet Avatar, OMNIC version 6.2 software, Golden Valey, MN, USA) from 4000 to 500 cm^−1^. The efficiency of the encapsulation was obtained indirectly by following the methodology in liquid phase by ultraviolet–visible (UV–Vis) spectroscopy (UV-240 LPC, Shimadzu, Tokyo, Japan) with UVProbe photometric software (version 2.43,Tokyo, Japan).

### 2.3. Characterization of Textile Finishing

The surface morphology of the complexes was examined by scanning electron microscopy (SEM) using a JEOL JSM (5610 instrument, Peabody, MA, USA). Microscopy was done on treated and untreated cotton and polyester fibers. For SEM, the textile articles were covered with gold using a sputtering chamber.

Attenuated total reflectance Fourier-transform infrared spectroscopy (FTIR-ATR, PerkinElmer, São Paulo, Brazil) was used to investigate the functional groups present on untreated and treated textile substrates. A PerkinElmer Frontier device with a resolution of 1 cm^−1^ was used with 64 accumulations in the intermediate infrared range from 650 to 4000 cm^−1^.

Studies of the controlled release of citronella essential oil from the textile substrates were determined in vitro in triplicate, as follows: after treatment with complexes and curing, cotton and polyester fabrics were placed in a thermostated bath at 37 ± 0.5 °C under low stirring conditions in a WNB14 shaker (Memmert, Schwabach, Germany) at a bath ratio of 1/100 to ensure semi-infinite conditions. Aliquots of 2 mL were extracted and filtered at predetermined times, and their absorbance was determined by spectroscopy at the ultraviolet maximum absorbance wavelength of 333 nm (oil), using a UV-240LPC spectrophotometer (Shimadzu, Tokyo, Japan). Adjustments were performed with OriginPro 8.5.1 software.

For statistical analysis, the data in triplicate are presented as mean ± standard deviation (SD). Statistical significance (*p* < 0.05) was determined by one-way analysis of variance (ANOVA) using OriginPro 8.5.1 (Northampton, MA, USA).

## 3. Results and Discussion

### 3.1. FTIR Study of βCD–Citronella Complexes

**Figure 4:**
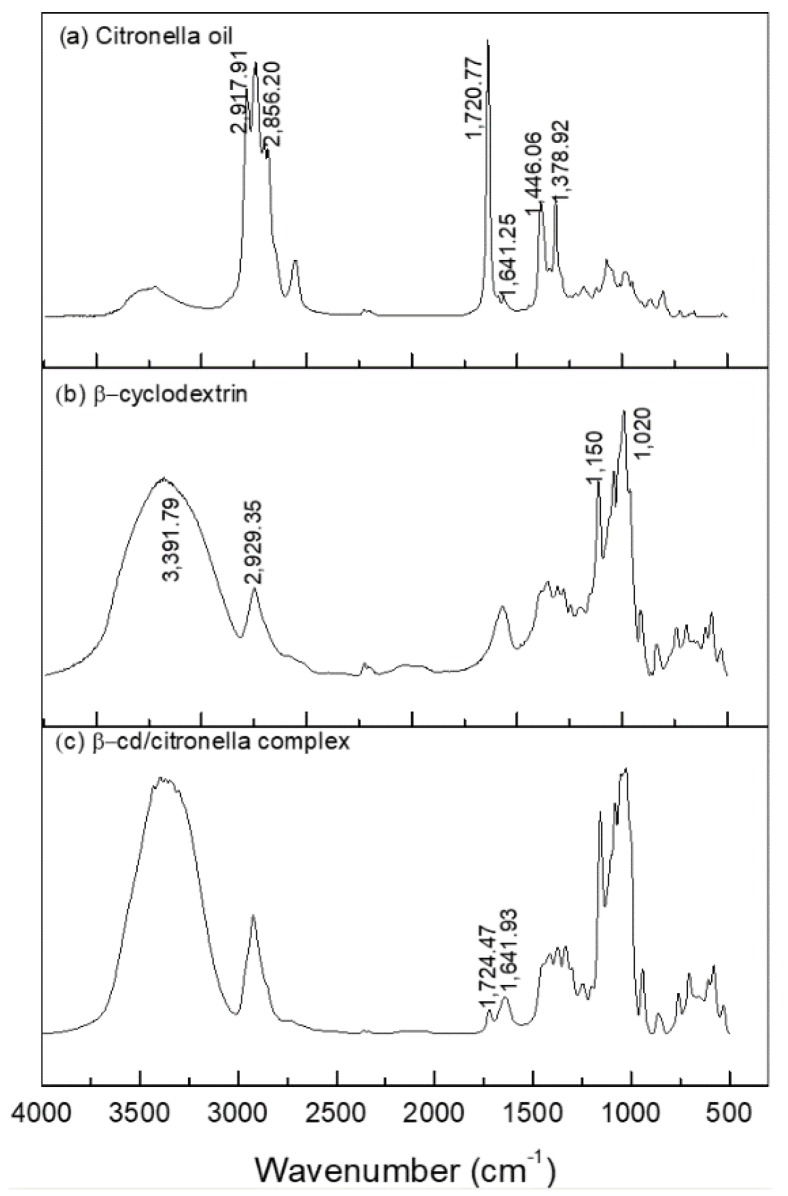
Spectroscopy in the infrared region of: (**a**) citronella oil, (**b**) *β*CD, (**c**) *β*CD–citronella oil complex.

The formation of the citronella–*β*CD complex was also confirmed by FTIR-ATR. The spectra in the infrared region are shown in Figure 4. 

Figure 4a shows that the spectrum corresponding to citronella allows for observation of the presence of the vibrational mode of CH carbonyl bonded aldehyde and C=O carbonyl bonded aldehyde [40], with bands corresponding to 2856.20 and 1720.77 cm^−1^, respectively. CH_2_ asymmetric stretching and CH_3_ asymmetric bending are also observed as bands in the regions 2917.91 and 1446.06 cm^−1^.

The spectrum of *β*-cyclodextrin showed prominent absorption bands (Figure 4b) at 3391.79 and 2929.35 cm^−1^. For the typical peak of the OH group, broadband indicates that it is part of hydrogen-bridge bonds. The peak in the region 2929.35 cm^−1^ shows the presence of aliphatic CH_2_ (secondary carbon) and at 1020 cm^−1^ shows a secondary alcohol stretch present in the CD molecule, as shown in Figure 6.

The FTIR spectra of the inclusion complexes of *β*CD–citronella obtained through the mechanical ultra-agitation method (Figure 4c) were compared to those of the citronella oil and *β*-cyclodextrin. Encapsulation of the oil in the interior of the cyclodextrin by intermolecular interactions makes the CD signals predominant [28]. Nevertheless, the FTIR spectrum obtained for the complex is a typical representation of a physicochemical interaction, a simple convolution of spectra [27]. Thus, there was no formation of new chemical bonds, and therefore no major effective changes in the spectra [41].

The formation of the complex can be confirmed by the bandwidth reduction in the 3391.79 cm^−1^ region, which, according to Aguiar et al. [42], is reduced by the interactions of the encapsulated molecules with CD changing it. 

On the other hand, from the FTIR spectrum of the inclusion complex it is possible to observe the presence of bands at 1720.77 and 1641.25 cm^−1^, the first characterized by the band of the C=O aldehyde carbonyl group and the second by the C=C alkene stretch, groups that are present in the citronella molecule. This result was found in the work of Santos et al. [40], in which they performed the inclusion of the essential oil of citronella in cyclodextrin.

### 3.2. Complexation Yield

To assess the yield of the complexation process, spectroscopy in the UV region of citronella oil in acetone solution was performed in order to detect the wavelength of maximum absorption (from 250 to 550 nm) using Equation (1):

(1) $$C_{\mathrm{oil}}=0.079+0.003abs_{333}$$

where *C*_oil_ is the oil concentration, mg mL^−1^, and abs is the absorbance (fractional %) at the wavelength of 333 nm.

Table 1 shows the angular and linear coefficient values found for the adjusted straight line and the determination of the coefficient value. 

From the values of the remaining concentrations of citronella oil after the complexation treatment, and in comparison with the initial amount, the yield of the process can be calculated. Table 2 shows the yield of the complexation process.

The average complexation yield of OC with *β*CD was 63.79 ± 0.38% (Table 2). Compared to other methods that use biopolymers for the encapsulation of citronella oil, as in the work of Bezerra et al. [7], in which the microencapsulation of the citronella essential oil was carried out using gelatin and gum arabic, an efficiency of 51.37 ± 3.33% was obtained. This result is shown to be lower than the efficiency when cyclodextrin was used. The results of application of the complexes on the textile substrates can be seen in Table 3.

The results indicate that at the end of the process, the presence of complexes in the textiles is observed, varying in accordance with the type of textile substrate. The item that presented a greater retention of complexes was the cotton fabric, 11.32 ± 0.71%; this value is superior to that found by Martin et al. [15], who applied *β*CD on nonwoven cotton fabrics. Martí et al. [43] showed that the differences between the percentages of retention of textile items (cotton and polyester) are related to possible interactions between the textile substrate and the finishing, due to the presence of hydroxyl groups that allow esterification by BTCA.

### 3.3. Morphology

**Figure 5:**
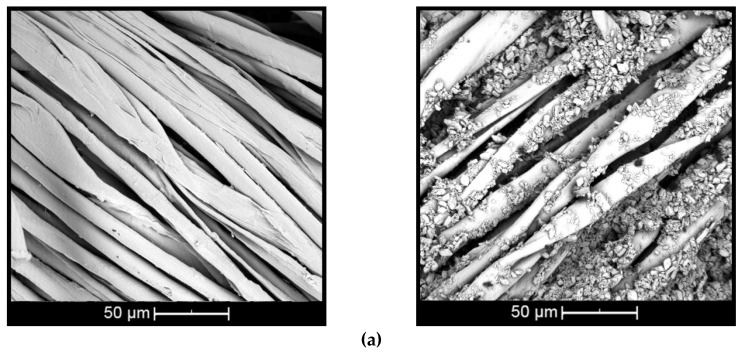

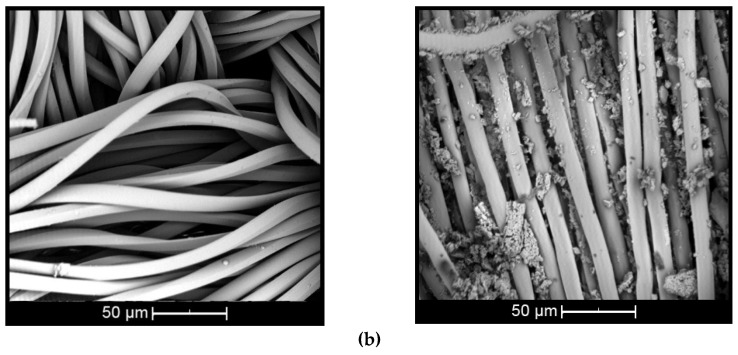
Scanning electron images of complex applied on: (**a**) cotton and (**b**) polyester (untreated textiles on left and treated on right).

The morphology of *β*CD oil complexes (wt % *β*CD and 10 wt % citronella oil) deposited on the surface of cotton and polyester by means of the esterification reaction using the BTCA crosslinking agent is presented in Figure 5. SEM analysis was done to prove the presence of complexes on the surface and observe the morphological changes of the treated and untreated textiles. 

The surface of the untreated cotton fiber (Figure 5a, left) shows disturbances and small twists [44], while the untreated polyester fiber (Figure 5b, left) presents cylindrical and smooth morphology [45]. When the fiber is treated with *β*CD–citronella complexes, there is a nonuniform distribution of complexes and the formation of some aggregates, both on the cotton and on the surface of the polyester fiber (Figure 5a,b, right). This can also be seen in the study by Mihailiasa et al. [8], which applied melatonin–cyclodextrin complexes for functionalization of the surface of cotton fabrics. The complexes applied to the textile cover its surface, enabling formation of the finish layer, as stated by Oliveira et al. [37].

These aggregates are formed by the esterification reaction between the OH group of the cyclodextrin and the COOH group of the BTCA, binding one cyclodextrin to another, as shown in the scheme of Figure 1. In the case of cotton fiber, there is still the interaction with the OH group, allowing the fiber coating [16,17,18]. In the case of polyester, the fiber is surrounded by the complexes, as shown in Figure 2, according to Blanchemain et al. [19], and a network of the complex is formed by coating the crosslinked polymer formed between the CD and the BTCA via the polyesterification reaction.

### 3.4. Application on Textile Substrate

**Figure 6:**
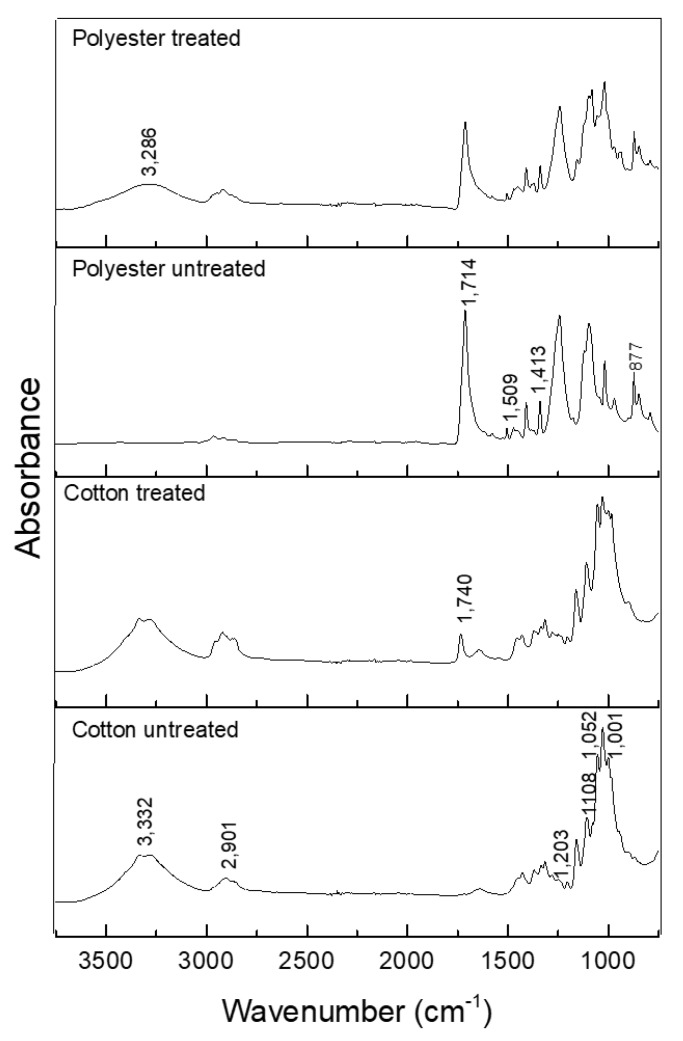
ATR-FTIR spectra of treated and untreated cotton and polyester textile.

Figure 6 shows a spectrogram in the infrared region of untreated textile items and those treated with CD–OC complex.

The following bands were observed in the spectrum for untreated cotton: 1001–1052 cm^−1^ vibration of 1,4 *β*-glycosidic linkage; 1.108 cm^−1^ asymmetric C–O stretch of pyran ring; and 1.203 cm^−1^ C–O stretch of alcohol in plane bend [46,47] (Figure 6).

In the treated cotton, the appearance of the band at 1740 cm^−1^, corresponding to the C=O stretch of the crosslink between BTCA and the hydroxyl group of cellulose, hydroxyl group of cyclodextrin, or both [8,14] (scheme of Figure 1), indicates the mechanism of esterification.

In the spectrum of the polyester fabric, the bands are at 877 cm^−1^, referring to the aromatic ring vibration of the benzene functional group; 1413–1472 cm^−1^ strong stretch of the C–O group of unsaturated and aromatic ester; 1509–1578 cm^−1^ aromatic skeletal vibration C=C. The analysis of the finishing using the *β*CD–OC complexes showed the presence of cyclodextrin; by comparison, Figure 5 shows the same characteristic bands [48]. There is the emergence of a band at 3.286 cm^−1^, characteristic of the OH wide band, present in the cyclodextrin, showing its deposition on the textile. As seen in the scheme shown in Figure 2, the complexes cover the fiber, which is also indicated in Figure 5b.

### 3.5. Release Kinetics of Citronella Essential Oil

In the present work, in vitro release experiments were performed on both treated and untreated cotton and polyester fabrics. In order to determine if the amount of citronella released from the fabrics could be modified by the treatment, mathematical adjustments were made to investigate the mechanism of drug release from the polymeric matrices.

Peppas and Korsmeyer [49] created a simple semi-empirical model that relates exponentially the release of the active principle with the time elapsed. This equation can be written as follows:

(2) $$\frac{M_{t}}{M_{\infty }}=K_{\mathrm{KP}}t^{n}$$

where $K_{\mathrm{KP}}$ is the Korsmeyer kinetics rate constant, which incorporates structural and geometric characteristics; and n is the release exponent, indicator of the mechanism, according to Table 4, related to the geometry of release [50]. Adjustments were performed with OriginPro 8.5.1. 

Thus, the model proposed by Korsmeyer-Peppas is generally applied to analyze the release of polymeric dosage forms when the release mechanism is not known, or when more than one type of release is involved [34,50].

The most used mathematical expression was derived from the Higuchi [53] equation for planar films:

(3) MtM∞=KHt12

where $\frac{M_{t}}{M_{\infty }}$ is the ratio between the amount of release of the active principle at each point of time *t* and *K*_H_ is the Higuchi constant.

The Higuchi model of release is based on Fick’s law for short values of the square root of time [34].

**Figure 7:**
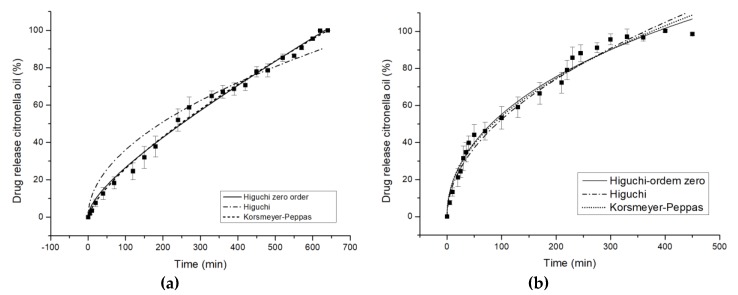
In vitro controlled release profiles of Higuchi zero order model, Higuchi model, and Korsmeyer-Peppas model in water at 37 °C for complex citronella essential oil applied on (**a**) cotton and (**b**) polyester.

The accumulative release kinetics profiles as a function of time are shown in Figure 7. The maximum amount of citronella oil released is achieved after 600–660 min for cotton and 300–360 min. for polyester treated with the complexes, while with untreated fabrics it is instantaneous.

The release profiles of cotton and polyester fabrics are illustrated in Figure 7, and the corresponding values of the quantitative approach of models are shown in Table 5. For the COT textile substrate, the value of exponent *n*, 0.7414, indicates an anomalous diffusion mechanism, as shown by Scacchetti et al. [29], who complexed thyme oil to cyclodextrin and applied it on cotton, yielding *n* = 0.620 ± 0.0220. An explanation for the anomalous mechanism is that cyclodextrin swells in water, which, according to Surathi and Karbhari [54], is seen as anomalous. One mechanism is interfering with, or affecting, the priority of the other. As the authors have shown previously [7], the chemical character of the fabric also strongly influences the delivery mechanism. In this specific case, the organic character of citronella oil makes it less attractive for the fiber chemical group. This means the control of the mass transfer mechanism would be controlled by CD interactions.

With respect to the release profile of polyester (Figure 7b), the value of exponent *n*, 0.4639, which is very close to 0.5, indicates a Fickian diffusion mechanism. The mechanism is different than in the case of COT. Here, the chemical similarities between the molecule to diffuse and the textile substrate make the mechanism totally dependent on the amount of citronella oil in the fabric, acting as the slab model proposed by authors in the mathematical approach.

It can be seen that the degree of hydrophobicity influences, in absolute terms, the value of exponent *n*.

## 4. Conclusions

The results of this study indicate that the inclusion of citronella oil in *β*CD can be obtained by high agitation and these complexes can be applied to textile articles by crosslinking agents. This interaction, between cyclodextrin and fibers was achieved by means of the esterification reaction using butane-1,2,3,4-tetracarboxylic acid (BTCA), as crosslinking agents. Free oil molecules and molecule complexes were obtained with the proposed methodology, as shown in the results of SEM, optical microscopy, and, mainly, FTIR. The prepared complexes had an efficiency of 63.79%.

The in vitro release kinetics study showed that the release mechanisms in PES and COT are different. That difference can be attributed to the index of hydrophilicity of each substrate and the mobility of polymeric chains in the microstructure of each one. The results of release kinetics clearly show the possibility of using these kinds of complexes to control the release of an active principle for long periods of time, indicating that the textile matrix directly influences the release mechanism.

The knowledge obtained from this study indicates that finishes using *β*CD complexes are effective, providing important insight for the development of biofunctional fabrics intended to fight mosquitoes, and textiles can contribute to this.

## Figures and Tables

**Table 1:** Values corresponding to the equation generated for calculating the concentration of citronella essential oil at 333 nm.

	Value	Error
Angular coefficient (a)	−0.0359	0.0267
Linear coefficient (b)	12.66	0.2920
Adjusted *R*^2^	0.9998	

**Table 2:** Yield of the complexation process of citronella essential oil by *β*-cyclodextrin.

Codes	Initial Concentration of Oil (mL mg^−1^)	Final Concentration Free of Citronella (mL mg^−1^)	Yield (%)
1	0.0588	0.02151	63.43
2	0.0600	0.02149	64.18
3	0.0588	0.02172	63.75
Mean Standard Deviation			63.79 0.38

**Table 3:** Results of the finishing process with complexes in cotton and polyester fabric.

Parameters	Cotton	Polyester
**Mass (g)**	0.212 ± 0.005	0.210 ± 0.017
Pickup (%) *	122 ± 0.5	122 ± 0.90
O.W.F. (%) **	11.32 ± 0.71	6.19 ± 0.91

* Pickup (%): theoretical percentage of product present after impregnation, wet fabric, with relation to the dry fabric; ** O.W.F. (%) (on weight of fibers): amount of product applied, calculated by mass difference between untreated dry fabric and after the finish.

**Table 4:** Release system related to Korsmeyer-Peppas exponent n based on geometry [51,52].

Surface	Cylinder	Sphere	Diffusion Mechanism
0.50	0.45	0.43	Fickian
0.50 < n < 1.00	0.45 < n < 0.89	0.43 < n < 0.85	Anomalous
1.00	0.89	0.85	Non-Fickian

**Table 5:** Model parameters for controlled release of complexed oil applied to cotton and polyester.

Model	Parameter	Cotton	Polyester
Higuchi	*R* ^2^	0.9540	0.9793
	*K*_H_	0.0360 ± 0.0009	0.0523 ± 0.0008
	*D*_f_ (10^−3^)	0.254 ± 0.0127	0.5370 ± 0.0164
Korsmeyer-Peppas	*R* ^2^	0.9951	0.9811
	*K*_KP_	0.0083 ± 0.0001	0.0640 ± 0.0072
	*n*	0.7414 ± 0.0202	0.4639 ± 0.0202

Note: *D* is (Dδ2) diffusion coefficient (min^−1^).
